# Vascular stiffening in aging females with a hypertension‐induced HIF2A gain‐of‐function mutation

**DOI:** 10.1002/btm2.10403

**Published:** 2022-10-03

**Authors:** Eugenia Volkova, Linda Procell, Lingyang Kong, Lakshmi Santhanam, Sharon Gerecht

**Affiliations:** ^1^ Department of Chemical and Biomolecular Engineering Johns Hopkins University Baltimore Maryland USA; ^2^ Institute for NanoBioTechnology, Johns Hopkins University Baltimore Maryland USA; ^3^ Department of Anesthesiology and Critical Care Medicine Johns Hopkins University School of Medicine Baltimore Maryland USA; ^4^ Department of Biomedical Engineering Johns Hopkins University School of Medicine Baltimore Maryland USA; ^5^ Department of Biomedical Engineering Duke University Durham North Carolina USA

**Keywords:** Arterial Stiffening, Extracellular Matrix, HIF, HIF2a, Hypertension, Hypoxia, Pulmonary Arterial Hypertension, Systemic Arterial Stiffening

## Abstract

Pulmonary arterial hypertension (PAH) is more prevalent in females than males; the causes of this sex difference have not been adequately explored. Gain‐of‐function (GOF) mutations in hypoxia‐inducible factor 2α (HIF2A) lead to PAH and thrombotic consequences in patients and mice. Additionally, multiple emerging studies suggest that elevated systemic arterial stiffening (SAS) occurs in PAH; this could have critical prognostic value. Here, we utilized a HIF2A GOF mouse model to determine how SAS can be used as a prognosticator in sex‐divergent PAH. We analyzed survival, vascular mechanics, and vascular phenotypes in young adult (8–16 weeks) and middle age (9–12 months) Hif2a GOF mice. We find that Hif2a heterozygous (HT) female mice, but not Hif2a HT male mice, exhibit poor survival, SAS upon aging, and decreased ability to withstand repeated physiological strain. Hif2a HT female mice also display thickening of the adventitial intima and increased collagen I and collagen III in all layers of the thoracic aorta. Our findings demonstrate differing PAH progression in female and male Hif2a GOF mice. Specifically, alterations in extracellular matrix (ECM) content led to vascular stiffening in aged females, resulting in poor survival. Moreover, we show that SAS emerges early in mice with PAH by coupling studies of vascular mechanics and analyzing vascular structure and composition. Importantly, we present a model for assessing sex differences in hereditary PAH progression and sex‐specific prognosis, proposing that aortic stiffening can be used to prognosticate future poor outcomes in PAH.

## 1 INTRODUCTION

Pulmonary arterial hypertension (PAH) is a multifactorial disease characterized by loss of compliance and remodeling of PAs, resulting in right heart failure and death. PAH progression correlates with vascular stiffening and remodeling, vasoconstriction, endothelial dysfunction, inflammation, and thrombosis.^1^, ^2^, ^3^, ^4^, ^5^ PAH‐associated arterial remodeling contributes to right heart failure via ventricular–vascular coupling.^6^, ^7^, ^8^ PAH‐associated remodeling is typically characterized by the thickening of the three structural arterial layers.^9^ This structural thickening is due to changes in the extracellular matrix (ECM) quantity and the composition of each layer.

The estimated incidence of PAH is 1.1–7.6 per million adults per year, with a prevalence of 6.6–26.0 per million adults per year.^10^, ^11^, ^12^ Neither idiopathic nor heritable PAH is sex‐blind; both predominately affect females.^13^, ^14^, ^15^ Interestingly, the balance of PAH incidence in the two sexes changes with age. The COMPERA European registry showed that the overall 1.8:1 female‐to‐male ratio fell to 1.2:1 when limited to patients older than 65.^16^ Moreover, PAH is most commonly diagnosed in women between the ages of 30–60, while males are often diagnosed with PAH at older ages.^17^ This asymmetric relationship concerning the time of initial diagnosis and incidence is inadequately understood. To address the gap in our understanding of the differences in prevalence and incidence of PAH in male and female patients, we aimed to examine PAH progression in the context of sex and age.

Accumulating evidence shows that PAH may also cause systematic arterial stiffness (SAS).^18^, ^19^ Multiple prior clinical studies have correlated increases in SAS with PAH patient disease progression.^20^, ^21^, ^22^ Specifically, increased vascular stiffness is positively associated with age and disease progression and negatively correlated with survival.^20^, ^21^, ^22^ Nonetheless, the prognostic value of vascular stiffness remains incompletely understood but points to the possibility of PAH as a cause of accelerated arterial aging or to the potential for vascular interdependency.^23^, ^24^ Thus, in the present study, we sought to determine if PAH pathogenesis accelerates the deterioration of systemic vascular mechanics with age and whether this links to mouse sex.

Hypoxia‐inducible factor (HIF) signaling plays a fundamental role in PAH pathogenesis.^25^ HIF activity is regulated in an oxygen‐dependent manner; degraded in normal oxygen conditions, HIFs stabilize and accumulate in hypoxia. Multiple mutations in the HIF2A gene associated with erythrocytosis have been identified, with some patients developing pulmonary hypertension.^26^, ^27^, ^28^, ^29^, ^30^ Specifically, missense mutations in H1F2A G537W impair HIF2a hydroxylation and have been shown to cause familial PAH.^30^ Mice with missense Hif2a G536W mutations display mutation‐dependent erythrocytosis and pulmonary hypertension.^31^, ^32^ Moreover, smooth muscle cells from mice and patients with missense H1F2A mutations exhibit increased stiffness and abnormal f‐actin fiber orientation.^32^

In the present study, we sought to determine whether SAS occurs in familial hypertensive aging female mice compared with male counterparts, thereby providing SAS as a prognosticator of PAH. We study the differences in the temporal evolution of HIF2A‐driven PAH‐associated ECM changes. Specifically, we examine whether this can be attributed to sex, a variable infrequently assessed in the PAH field. We leverage the HIF2A GOF mouse model, previously established consistent with hereditary Hif2a G537R, and we couple survival, circumferential tensile testing (CTT), gene expression, and immunohistochemical (IHC) analyses to probe the contributions of collagens I, III, and elastin to physiological mechanical properties of the hypertensive thoracic aorta.^32^

## 2 RESULTS

### 2.1 Female Hif2a HT and HO mice display characteristics of PAH and erythrocytosis

Previous studies have shown that mutations in G537 of the HIF2A gene cause familial hypertension.^26^, ^31^, ^32^ Thus, we focused on a Hif2a G536W knock‐in mutation in a C57BL/6 background mouse.^30^ While prior studies have extensively characterized and assessed this mouse model as having pulmonary hypertension (RV pressure > 60 mm Hg in HO) and erythrocytosis, they have not confirmed the disease presentation of the female mice.^31^, ^32^ We mated Hif2a^G536W/+^ (heterozygote; HT) mice and obtained both Hif2a HT and Hif2a^G536W/G536W^ (homozygote; HO) mice. Hif2a^+/+^ (wild‐type; WT) littermates served as controls. However, all HIF2A gene mutations in humans are heterozygous^31^; the homozygous mutation is lethal in humans.

First, we measured the mice's red blood cell (RBC) counts. The RBC count for aged female mice increased in a mutation‐dose‐dependent (Figure S1A). We then measured Fulton Index and normalized heart weight for combined middle‐aged and early aging female WT, Hif2a HT, and Hif2a HO mice. We observed increased normalized heart weight in aged female Hif2a WT mice (Figure S1B‐C). We next measured hematocrit and hemoglobin and marked increases in aged female Hif2a HT and Hif2a HO mice (Figure S1D,E). We found no statistically significant differences in the groups when measuring the white blood cell, platelet, and immune cell counts (Figure S1F,G). We performed the same analysis on aged male WT, Hif2a HT, and Hif2a HO mice and found similar trends except for increased statistical significance in the lymphocyte counts (Figure S2).

### 2.2 Aging female Hif2a heterozygote and homozygote mice exhibit higher mortality than their male genetic counterparts

**FIGURE 1:**
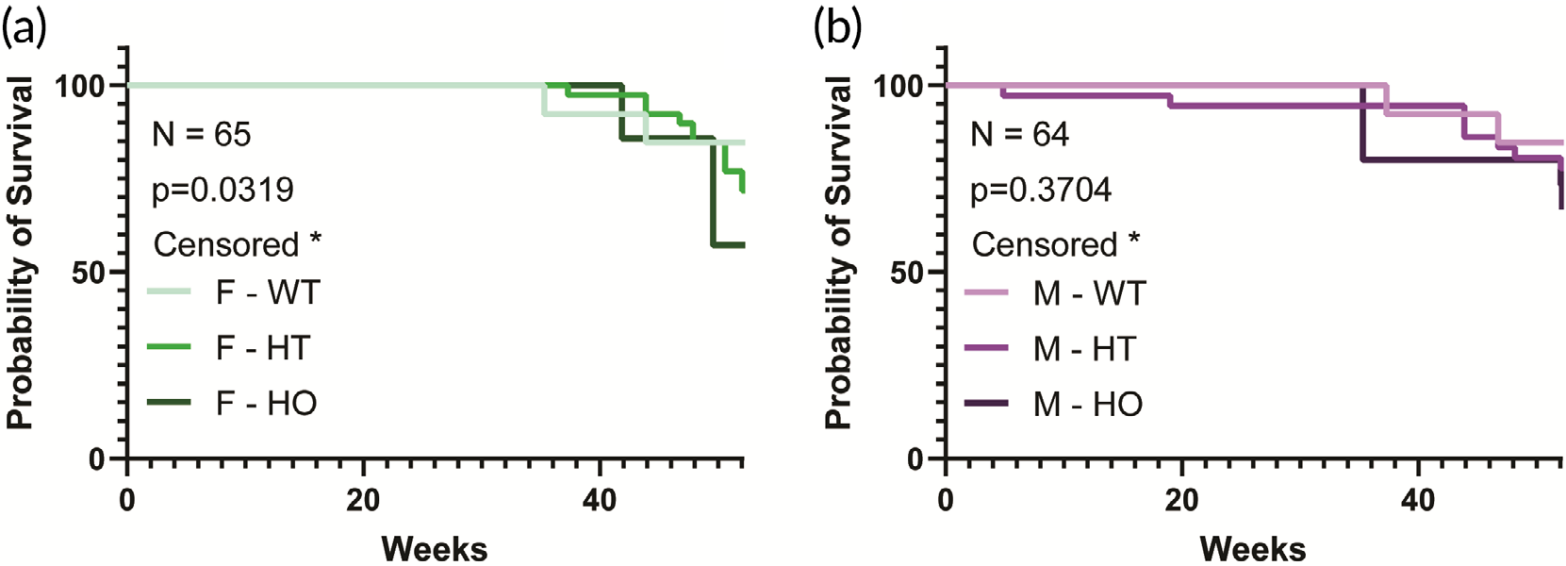
Sex differences in survival rates in WT and Hif2a mice with aging. Kaplan–Meier survival analysis for WT, Hif2a HT, and Hif2a HO (a) female (*N* = 65) and (b) male mice (*N* = 64). Significance level is set at *p < 0.05 for a log‐rank (Mantel‐Cox) test.

After confirming PAH in the mouse model, we performed aging studies to elucidate changes in the distribution of genotypes and their likelihood of survival to 12 months. Consistent with prior studies, Hif2a HO mice were born at a lower frequency than expected according to the Mendelian genetics (data not shown), suggesting the Hif2a HO mice have a survival disadvantage from birth.^31^ Female mice exhibited mutation dose‐dependent differences in their mortality trends; a phenomenon that was not seen for the male mice (Figure 1). When directly compared, Hif2a HT female mice trended toward higher mortality than Hif2a HT male mice compared to their WT counterparts (Figure S3). In addition, our data suggest that female Hif2a HT and HO, mice have a disadvantage in survival with aging. Thus, we focused our study on female mice. Given the precipitous increase in mortality in the PAH group at ~13 months of age when compared with WT littermates, we defined the age groups of interest as being: young adult for 8–16 weeks, early middle age for 4–8 months, middle age for 9–12 months, and early aging for 13 months+. Our studies focused on a direct comparison of the young adult and middle age groups, as survival in the early aging group provided limited subjects for comparison.

### 2.3 Middle age female Hif2a HT mice have stiffer thoracic aorta than their young adult counterparts

**FIGURE 2:**
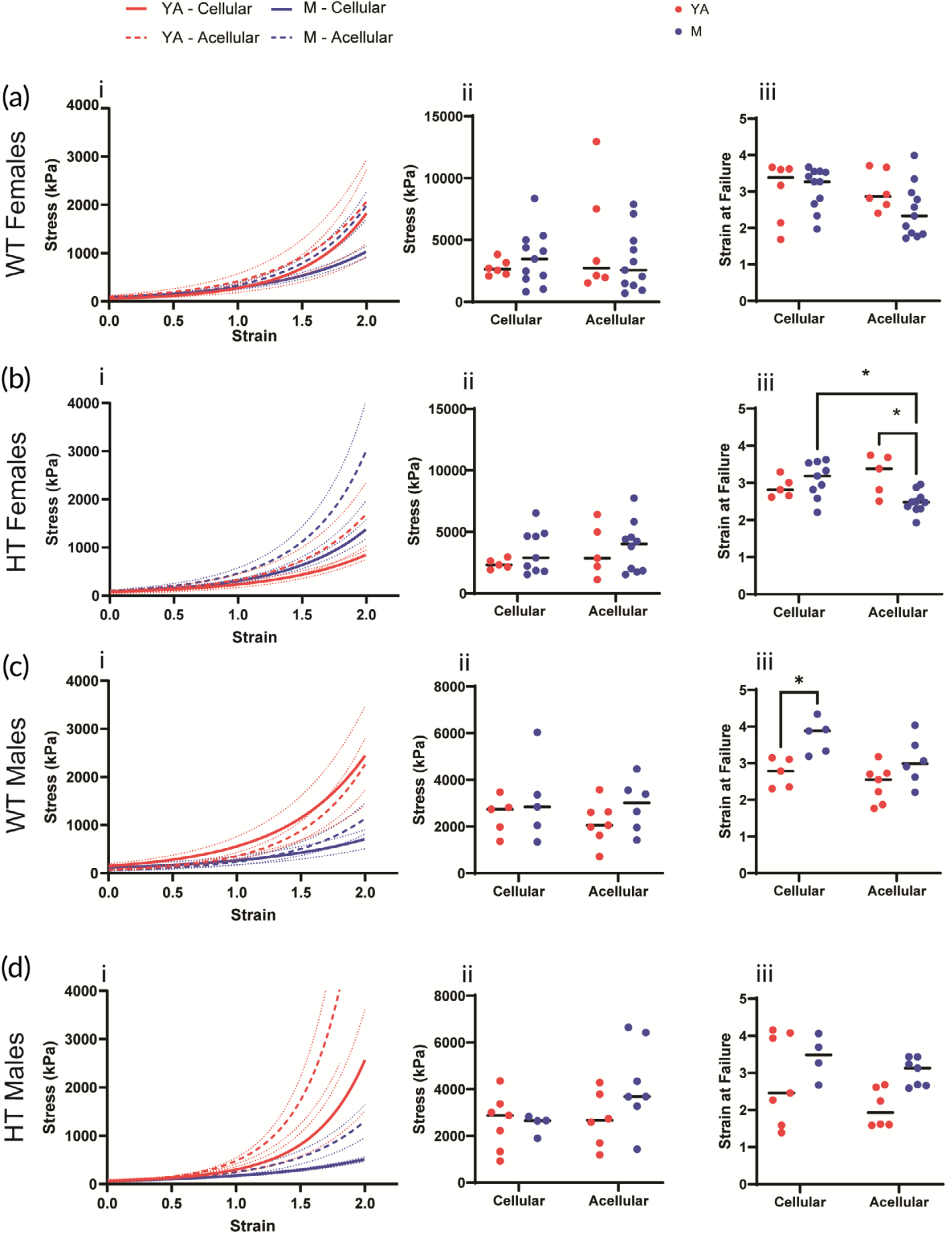
Female Hif2a HT mice exhibit compromised vascular mechanics because of aging‐dependent disease progression. (a) Exponential fits of tensile curves of circumferential tensile testing (CTT) of young adult (YA, 8–16 weeks) and middle age (M, 9–12 months) female WT thoracic aorta segments (i), ultimate tensile stress (ii), and strain at failure (iii) (*N* = 6–11, *n* = 2–4). (b) Exponential fits of tensile curves of CTT of young adult (YA, 8–16 weeks) and middle age (M, 9–12 months) female Hif2a HT thoracic aorta segments (i), ultimate tensile stress (ii), and strain at failure (iii) (*N* = 5–10, *n* = 2–4). (c) Exponential fits of tensile curves of CTT of young adult (YA, 8–16 weeks) and middle age (M, 9–12 months) male WT thoracic aorta segments (i), ultimate tensile stress (ii), and strain at failure (iii). (*N* = 6–7, *n* = 2–4). (d) Exponential fits of tensile curves of circumferential tensile testing (CTT) of young adult (YA, 8–16 weeks) and middle age (M, 9–12 months) male Hif2a HT thoracic aorta segments (i), ultimate tensile stress (ii), and strain at failure (iii) (*N* = 7, *n* = 2–4). Solid lines indicate cellular segments, dashed lines indicate acellular segments, and dotted lines indicate bounds of SEM. Significance level is set at *p < 0.05 for a two‐way ANOVA (multiple comparisons performed using Tukey's test).

Emerging studies link SAS to PAH. Therefore, we next sought to examine whether female WT and Hif2a HT mice had any alterations in their response to functional tensile stress with aging; thus, we examined the response of the thoracic aorta using CTT. We focused on the young adult (8–16 weeks) and middle‐aged (9–12 months) age groups, which correlate approximately to young adolescence/young adulthood (13–20 years) and middle ages (30–40 years) in humans, respectively.^33^ To assess the contribution of the ECM to the mechanical properties, we decellularized the thoracic aortas and measured the mechanical properties of cellular and acellular segments of the same thoracic aorta. CTT data were represented by the equation S = α exp (βλ), where α and β are constants determined by least squares curve fitting. We found that middle‐aged female Hif2a HT mice exhibit vascular stiffening, as evidenced by the left shift in the stress versus strain curve (Figures 2ai,bi and S4A,B). Aging female WT mice did not exhibit a similar trend, suggesting that the vascular stiffening phenotype is specific to the PAH mice. We examined the maximum tensile stress for female WT and Hif2a HT mice and found no statistically significant differences (Figure 2aii,bii). Finally, we examined strain at failure (Figure 2aiii,biii) and found a dramatic decrease between female Hif2a HT acellular young adult and middle age segments' strain at failure, suggesting that thoracic aortas of aged female Hif2a HT mice experience more significant changes in their ECM and that these changes dramatically impact their ability to handle physiological strain (Figure 2biii). The thoracic aorta of middle‐aged female Hif2a HT mice also exhibited statistically significant differences between the strain at failure of their cellular and acellular segments (Figure 2aiii,biii). The variability in the middle age female WT mouse cellular and acellular segments was greater than that of any of the other vessel subgroups, suggesting that aging also carries the consequences of increased variance in the composition and subsequent contribution of the ECM to vascular mechanical properties (Figure 2biii).

**FIGURE 3:**
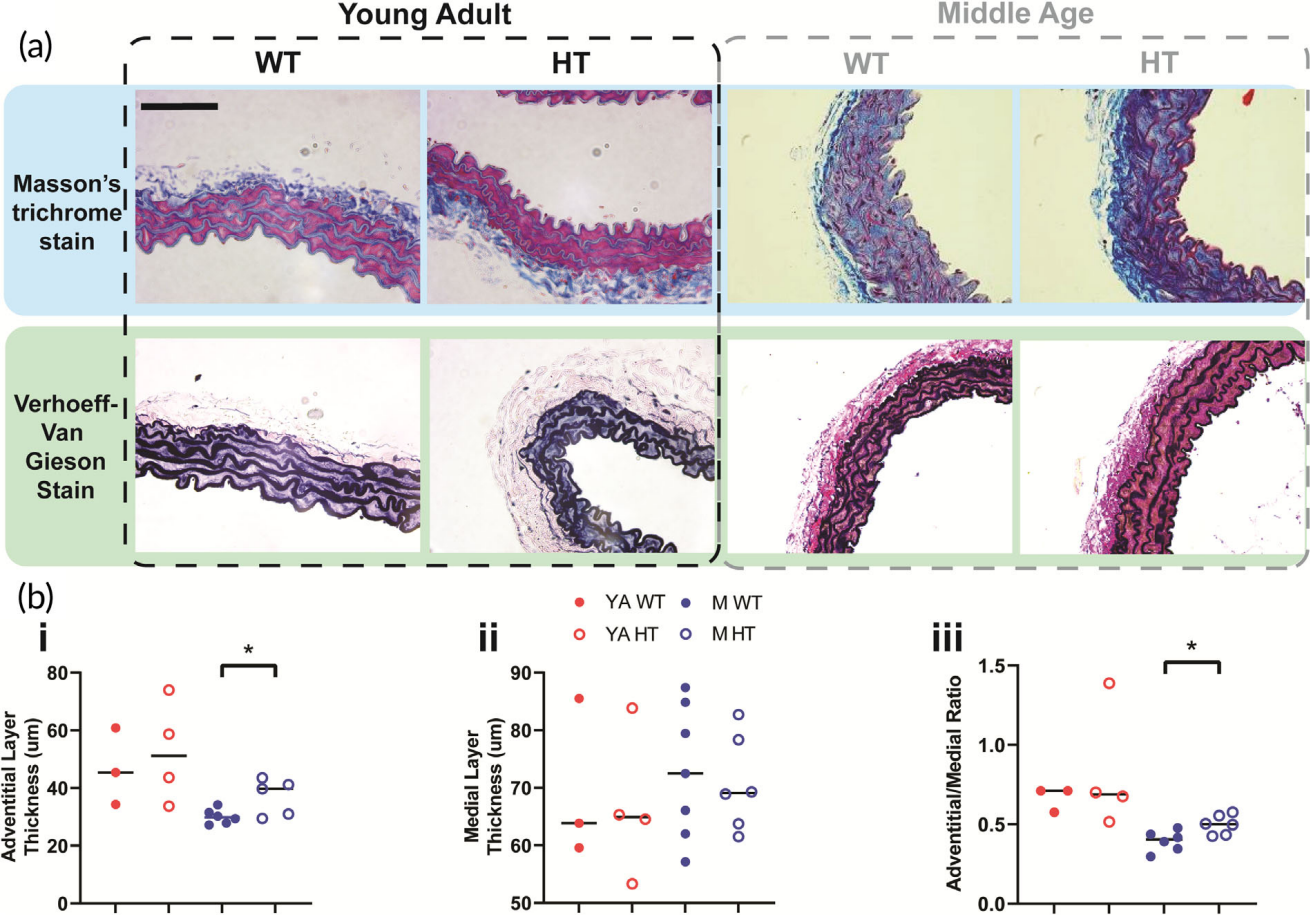
Hif2a HT middle age female mice exhibit a thicker adventitial layer than their wildtype counterparts. (a) Representative 40× histological images of Masson's Trichrome (MAS) and Verhoeff‐Van Gieson (VVG) stained of young adult (YA, 8–16 weeks) and middle age (M, 9–12 months) female WT and Hif2a HT mouse thoracic aorta. Scale bar is 75 μm. (b) Quantification of histological images of adventitial layer thickness (i), medial layer thickness (ii), and adventitial/media thickness ratio (iii) (*N* = 3–7). Significance level is set at *p < 0.05 for a two‐way ANOVA (multiple comparisons performed using Tukey's test).

To adequately assess sex differences in how PAH evolves with HIF2a GOF, we performed the preceding experiments with male mice. Here too, we decellularized the thoracic aortas and measured cellular and acellular segments of proximal aortic segments. We found that middle age male WT and Hif2a HT mice are less capable of withstanding repeated physiological stresses than their young adult counterparts, evident by the right shift in the stress versus strain curve (Figure 3ci,di). We also examined the maximum tensile stress of the thoracic aorta from male WT and Hif2a HT mice and found no statistically significant differences (Figure 3cii,dii). Finally, we examined strain at failure. Interestingly, we found statistically significant differences between young adult and middle age cellular segments of male WT, a difference we did not see in the female counterparts (Figure 3ciii).

### 2.4 Middle age female Hif2a HT mice exhibit thoracic aorta adventitial layer thickening compared to WT counterparts

Next, we examined the differences in the ECM by immunohistochemistry (IHC) analysis of thoracic aortic rings. As the middle age Hif2a HT female mice exhibited vascular stiffening and statistically meaningful differences in their strain at failure compared to young adult Hif2a HT female mice, we focused on these two age groups. Littermate female WT counterparts served as controls. We completed Masson's trichrome stain (MAS) and Verhoeff‐Van Giesson (VVG) staining and quantified the thickness of the adventitial layer, medial layer, and the adventitial/medial layer thickness ratio for young adult and middle age female WT and Hif2a HT mice (Figure 3a,b). Middle‐aged Hif2a HT females, but not young adult Hif2a females, exhibit a statistically significant difference in the adventitial layer thickness and the adventitial/medial layer thickness ratio compared to their age‐matched WT counterparts (Figure 3bi,iii). No differences in the medial layer thickness were observed (Figure 3bii). We further quantified the characteristics of the elastin in the medial layer, including the number of lamellae, lamellar thickness, and interlamellar distance (Figure S5). While young adult and middle‐aged mice had different lamellar thickness, no statistically significant differences were found between the WT and Hif2a HT mice. Thus, we further focused on the major ECM components of the adventitial layer, the collagens, and their differences across our groups of interest.

### 2.5 Collagen I and collagen III accumulate in the aortas of middle age female HT mice compared to young adult female counterparts

**FIGURE 4:**
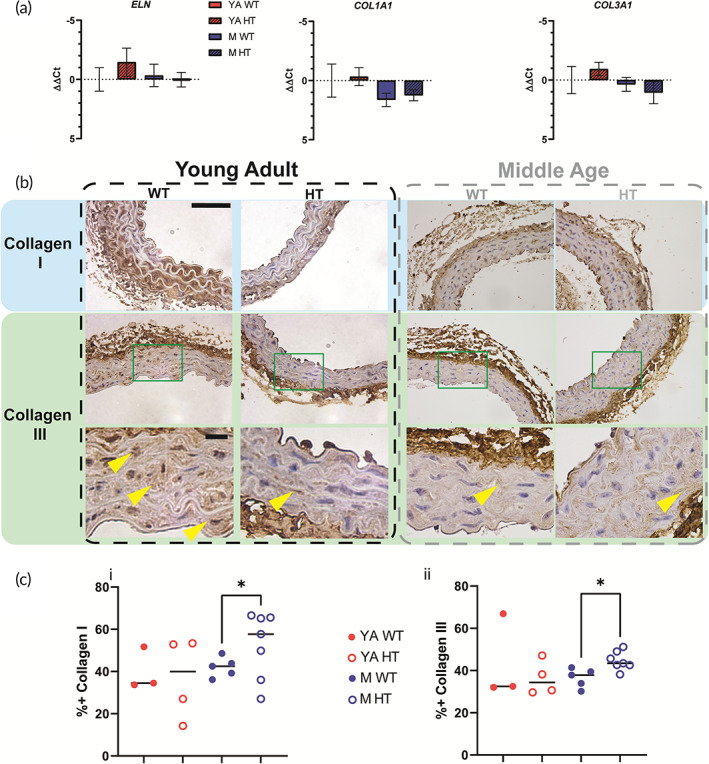
Middle age female Hif2a HT mice exhibit increased collagens I and III compared to wild‐type counterparts. (a) qRT‐PCR of relative expression of ELN, COL1A1, and COL3a1 transcripts in young adult (YA, 8–16 weeks) and middle age (M, 8–12 months) female WT and Hif2a HT mice (*N* = 3–4, *n* = 2). (b) Representative 40× IHC images of collagen I and collagen III in young adult (YA, 8–16 weeks) and middle age (M, 8–12 months) female WT and Hif2a HT mice (*N* = 3–7, *n* = 10–40). Bottom row are high‐magnification of the boxed areas for collagen III (green squares) (i) top scale bar is 50 μm, bottom scale bar is 10 μm. Yellow arrows mark collagen + regions of the medial layer. (c) Percentage of vessel area positive for collagens I and III + area (ii) (*N* = 3–7, *n* = 20–40). Significance level is set at *p < 0.05 for a *t*‐test (Kolmogorov–Smirnov comparison).

First, we examined differences in the ECM components by performing qRT‐PCR on mouse abdominal aortas (Figure 4a). We did not find any statistically significant differences in levels of ELN, COL1A1, and COL3A1 transcripts. We next evaluated collagens I and III accumulation by performing IHC for collagen I and collagen III on young adult and middle age WT and HT female mouse thoracic aortas. We found that Hif2a HT female mice have more collagens I and III than WT female mice (Figure 4b; Figure S6).

## 3 DISCUSSION

PAH is more prevalent in female patients than in male patients. While the causes of this sex difference have not been adequately explored, clinical database analyses have suggested several potential avenues.^34^, ^35^, ^36^ Researchers have found that female PAH positively correlates with patient use of prescription weight‐loss drugs, recreational drugs, and oral contraceptive pills.^17^ Researchers have also suggested that endogenous sex hormones, specifically 17β oestradiol and its metabolites, could cause increased female PAH incidence.^37^ Finally, some studies hypothesize that the higher incidence of autoimmune diseases (including systemic sclerosis, systemic lupus erythematosus, rheumatoid arthritis, Sjogren's syndrome, thyroiditis) in females, when further associated with PAH, has the potential to increase inflammation and to drive disease progression.^38^ However, these hypotheses do not adequately resolve why female PAH incidence is higher.^38^

Recent studies point to elevated SAS in PAH patients. This suggests that arterial aging may be accelerated in patients suffering from PAH and contribute to a further increase in cardiovascular risk. An intriguing possibility is whether elevated stiffening can be used as a prognostic for disease severity or progression. Thus, our study focused on resolving how hereditary PAH pathogenesis modulates systemic vascular mechanics with age and whether this links to the mouse sex. We first observed that female Hif2a HT and Hif2a HO mice are less likely to survive at 12 months. This effect is mutation‐dose‐dependent; female Hif2a HO and HT mice had poorer survival than female Hif2a WT mice. We confirmed that female and male Hif2 HT and Hif2a HO mice each exhibit the hallmarks of PAH and erythrocytosis. When all heterozygous groups were compared, only female HT mice exhibited SAS due to aging. This was evidenced in the CTT data, where the thoracic aorta of aging female Hif2a HT mice display stiffening, evident by the left shift in the stress versus strain curve. Strain at failure of acellular aortic segments was markedly reduced with age in the female Hif2a HT mice, indicating increased brittleness and a compromised ability to withstand repeated physiological strain in the aged vessels. The male Hif2a HT mice did not exhibit a right shift in the stress–strain curve; aortic stiffening, evident in female Hif2a HT mice, was absent in males. Additionally, consistent across multiple age groups, menstrual status did not confer protective effects.

We then examined the adventitial and medial layers of the thoracic aorta. Middle age female Hif2a HT mice exhibited thickening in their adventitial layer that is not seen in young adult female mice of either genotype. Surprisingly, we did not see any RNA transcript level differences between the female, young adult, and middle age WT and Hif2a HT mice. When we further examined middle age and young adult female Hif2a HT and WT mice for expression of collagens I and III, we found that middle age female Hif2a HT mice, compared to WT counterparts, have significantly greater collagen I and III deposits throughout their vessel architecture. This trend was also present in the younger female HT mice but to a lesser effect. Moreover, both middle age and young adult Hif2a HT mice exhibited a high degree of heterogeneity in their collagen I content, which may cause the large variability observed in vessel mechanics. Overall, collagens I and III depositions were both age and genotype dependent. Consequently, we conclude that elevated ECM deposition is the mechanism responsible for Hif2a HT female mouse aortic stiffening. Taken together, these findings suggest that accelerated aging of the systemic circulatory system and aortic stiffening indicate a poorer prognosis for survival.

Prior clinical studies have reported accelerated SAS associated with PAH; however, the biological foundations for this phenomenon remain undetermined and include systemic inflammation, main PA distension leading to irregular systemic hemodynamics, and pulmonary‐vascular oxygen and mechanical interdependency.^18^, ^19^, ^23^, ^24^ Prior research has also shown that in the systemic circulation, SMC stiffness can compensate for a more compliant ECM.^39^, ^40^, ^41^ We propose that this mouse model be utilized as a cell source for future *in vitro* studies to assess the mechanical contributions of sex‐differentiated SMCs to vascular mechanics; we predict that ECM and cell‐based changes are combinatorial in their functional results. These *in vitro* studies could also continue addressing unexplored sex‐specific PH differences. The Hif2a mutation compromises ECM strength with altered deposition of collagens leading to the lowered strain of failures. Further studies focused on matrix remodeling enzymes, including enzymes implicated in both crosslinking and degradation of the ECM, would further resolve the pathways driving the vascular functional decline.

We acknowledge that our study has limitations. Due to the structure and the already long timescale of the study, our Kaplan–Meier curves do not exceed 12 months. We do not address the cause of death in female PAH mice. We did not establish the causality between the Hif2a mutation and collagen deposition in the systemic vasculature. We also did not delve into sex‐specific differences in PAH cells. Examining the differences between male and female Hif2a HT and HO ECs and SMCs *in vitro* could elucidate additional causes for sex‐divergent disease progression. While we show that ECM compositional changes underlie the stiffening phenotype, the specific mechanism remains to be elucidated. In this context, further examination of the contributions of crosslinking enzymes, such as lysyl oxidase and lysyl oxidase homologs 1 and 2, and matrix metalloproteases (MMPs), such as MMP‐1, ‐2, and ‐14, might clarify matrix‐remodeling differences between male and female Hif2a HT mice and set the direction for available therapeutic solutions. Finally, delving into measurements of endogenous sex hormones in the blood and serum of male and female Hif2a HTs over time could further clarify disease progression and its mediators.

In summary, using the Hif2a GOF mice, we demonstrate different physiological consequences and PAH progression in female and male mice. This phenomenon recapitulates the higher prevalence and incidence of hereditary PAH in female patients. Moreover, we show that SAS emerges early in mice with PAH by coupling studies of vascular mechanics, using circumferential tensile testing, and analyzing vascular structure and composition. This is exacerbated in females when compared with age‐matched males. Aortic stiffening in PAH females is caused by alterations in ECM composition, leading to accelerated aging and increased mortality, suggesting that aortic stiffening could prognosticate poorer outcomes. Further, these findings will provide a model for assessing sex differences in hereditary PAH progression and therapeutic efficacy.

## 4 METHODS

### 4.1 Animal model

All animals used in these studies were maintained under protocols approved by the Animal Care and Use Committee at Johns Hopkins University School of Medicine. Hif2a HT mice were initially generated by the laboratory of Professor Frank Lee.^31^ Mating age‐matched nonlittermate Hif2a HT mice generated subsequent WT, Hif2a HT, and Hif2a HO mice. See Table 1 for details of the major resources used in these studies. Polymerase chain reactions (PCR) on young adult tail DNA were used to identify genotype, using primers for G536W. Mice were aged until they fit one of the predetermined age ranges, being a young adult (8–16 weeks), early middle age (16 weeks‐9 months), middle age (9–12 months), and early aging (13 months+). Animals were housed on a 12‐h light/dark cycle and were given access to food and water ad libitum. Animals were massed, sacrificed, and tissues were immediately collected for further experiments.

**TABLE 1:** Major resources table

Reagent/resource	Source	Identifier
Animals		
*Mus musculus*	Frank Lee^31^	C57/BL6, Male and Female
Antibodies		
Collagen I	Novus	Cat#: NB600‐408
Collagen III	Abcam	Cat#: ab7778
Chemicals		
Trizol reagent	LifeTech	Cat#:15596018
Ammonium hydroxide (NH_4_OH)	Sigma‐Aldrich	Cat#: 221228
Sodium dodecyl sulfate (SDS)	Bio‐Rad	Cat#: 1610301
Formaldehyde, 37 wt%	Fischer Scientific	Cat#: F79‐500
Critical commercial assays		
TaqMan Gene Expression Master Mix	Thermo Fischer Scientific	Cat#: 4369016
Dual Endogenous Enzyme Block	Agilent Technologies	Cat#200389‐2
ImmPRESS HRP anti‐rabbit IgG polymer detection kit	Vector Laboratories	Cat# MP‐7401
ImmPACT DAB HRP substrate	Vector Laboratories	Cat# SK‐4105
Oligonucleotides		
ACTB (Mm02619580_g1)	Thermo Fischer Scientific	Cat# 4453320
ELN (Mm00514670_m1)	Thermo Fischer Scientific	Cat# 4453320
COL1A1 (Mm00801666_g1)	Thermo Fischer Scientific	Cat# 4453320
COL3A1 (Mm00802300_m1)	Thermo Fischer Scientific	Cat# 4453320
Software and algorithms		
ImageJ	Kevin Eliceiri^42^	ImageJ
Excel	Microsoft	Excel
MATLAB	Mathworks	MATLAB
Prism Version 9.1.2	GraphPad Software Inc.	PRISM

Sex‐matched Hif2a HT and Hif2a HO mice were compared to their respective controls, the WT mice. Each individual mouse was treated as an experimental unit and is labeled as *N*# in the figure legends; all cases for *n*# refer to technical replicates. The number of experimental units allocated to each group at each portion of the study is detailed in the figure captions. For the CTT testing, the number of experimental units used was determined by performing a power analysis and setting an 80% confidence interval. While a 90% confidence interval would have been preferred, it required prohibitively large quantities of experimental units for some of the categories. For the Kaplan–Meier curves, the number of experimental units was determined based on colony size and available statistics after 2.5 years. All healthy experimental units that did not have other underlying medical problems were included in the experiment. Unhealthy experimental units that significantly deviated from the standard were excluded, some of the reasons why experimental units were excluded are obesity and anal prolapse. Randomization was not used during any part of the testing. For the cofounding variable of cage position on a rack, all mice tested during the same week, irrespective of the analysis, were stored on the same shelf of a rank and with the same proximity to the light for a minimum of 2 weeks. All researchers were aware of group allocation at all stages of the experiment and no blinding was conducted.

### 4.2 Kaplan–Meier analysis

We used a total of *N* = 126 mice for Kaplan–Meier curves. Of these, female WT *N* = 13, female Hif2a HT *N* = 39, female Hif2a HO *N* = 13, male WT = 13, male Hif2a HT = 36, male Hif2a HO = 15. Mice were tracked from approximate birth until their natural biological death or were censored prior to natural biological death if used for other terminal experiments. Log‐rank (Mantel‐Cox) tests were performed in GraphPad Prism 9 to evaluate the statistical significance.

### 4.3 Heart mass and complete blood cell count

Prior to vessel extraction and cleaning, blood was collected via cardiac puncture. Blood was collected in EDTA tubes, stored at 4°C for a maximum of 30 min, and a complete blood cell count (CBC) was performed by the Johns Hopkins University Phenocore, Phenotyping, and Pathology Core. After vessel extractions were complete, the heart was dissected on a surgical microscope, and components were massed. Fulton's index was defined as the weight of the right ventricle divided by the combined weight of the left ventricle and the septum.

### 4.4 Tensile testing

Circumferential tensile testing was performed following protocols described previously^43^, ^44^ with minor modifications.^45^, ^46^ The thoracic aortas were harvested, cleaned, and cut into 1.25–1.5 mm length segments. The length of each segment was imaged using a microscope set to a magnification of ×4. The length was measured using Image J software (NIH). The rings were then mounted onto pins on an electromechanical puller (DMT560). After appropriate system calibration and pin alignment, the pins were separated using an electromotor at a 50 μm/s to apply radial force onto the specimen until breakage. Displacement and force were continuously recorded. The thickness of the intimal and medial layers (t) and stress‐free lumen diameter (*D*_*i*_) of representative samples were measured from concentric rings cut adjacent to the pulled segments, imaged at ×4 magnification, and quantified using Image J software. Engineering Stress (S) was calculated by normalizing force (F) to the initial stress‐free area of the specimens (S=F/(2t*L)). Engineering strain (λ) was calculated as the displacement ratio to the initial stress‐free diameter. The raw data was processed in Excel (Microsoft) and MATLAB (MathWorks), where an exponential fit, using least‐squares fitting, was applied to the tensile testing data. All vessel segments that did not comply with the exponential fit with a minimum coefficient of determination, *R* ^2^ of 0.95 were excluded from the exponential fit curves and from the raw data set. All calculations were done using Excel (Microsoft) and MATLAB (MathWorks).

### 4.5 Thoracic aorta ring decellularization

The thoracic aorta rings were decellularized as published previously.^47^ Briefly, after cleaning, half of the rings were decellularized by washing in 50 mM NH_4_OH and 0.2% sodium lauryl sulfate (SDS) on an orbital shaker for 3 h. Three 30‐min washes in PBS followed decellularization. Testing of all thoracic aorta rings was performed in Ca^2+^‐free Krebs solution.

### 4.6 qRT‐PCR

Total RNA was isolated from the abdominal aorta using TRIzol (Invitrogen) according to the manufacturer's instructions.^48^ Extracted total RNA was quantified using an ultraviolet spectrophotometer and validated for having no DNA contamination. RNA was transcribed using reverse transcriptase M‐MLV and oligo(dT) primers (Promega, Madison, WI). The TaqMan PCR step was performed using TaqMan Universal PCR MasterMix and Gene Expression Assay (Applied Biosystems, Foster City, CA) for genes outlined in the text. The PCR step was performed with a StepOne Real‐Time PCR system (Applied Biosystems) according to the manufacturer's instructions. The relative expressions of these genes were normalized to the ActB amount in the same sample by using the manufacturer's ΔΔCT method. The comparative method was used to calculate the amplification differences between different samples for each primer set.

### 4.7 Histology, IHC staining, and quantification

For histological sectioning and staining, harvested thoracic aortas were fixed in 4% paraformaldehyde in buffered PBS for 24 h. Masson's Trichrome (MAS) and Verhoeff van Giesson (VVG) staining were performed by the Johns Hopkins University Reference Histology Core. IHC staining was performed as previously described using collagen I (Novus NB600‐408, 1:100) and collagen III (Abcam ab7778, 1:500). Stained tissues were imaged with an upright microscope (Nikon Accuscope 3000, DS‐F12).^49^ Image analyses were performed using MATLAB (MathWorks) and ImageJ (NIH).

The medial and adventitial layer thickness was measured on MAS‐stained slides by measuring the length of each layer perpendicular to approximate vessel curvature. Ten measurements were taken per 40× image, and a minimum of 10 40× images were averaged per animal (*n* = 10+). Elastin lamellar thickness, interlamellar distance, and the number of lamellae were measured on VVG‐stained slides by measuring the length of each object perpendicular to approximate vessel curvature. Ten measurements were taken per 40× images, and a minimum of 10 40× images were averaged per animal (*n* = 10+), except for the number of lamellar layers, which was measured in one location per 40× image.

Collagen I and collagen III staining analysis was performed using a custom MATLAB code based on the MATLAB internal “Color Thresholder” app and normalized to the total area of the vessel in each 40× image. Cohorts were protein and age matched to select ideal +positive thresholder values.

### 4.8 Statistics and reproducibility

For all experiments, “*n*” denotes technical replicates while “*N*” represents biological replicates. The number of technical replicates and biological replicates varies depending on the experiment and is noted specifically in each figure legend. Two‐tailed *t*‐tests or ANOVA were performed to determine significance. All graphs were drawn using GraphPad Prism 9. Significance levels were set at *p < 0.05, **p < 0.01, and ***p < 0.001.

## AUTHOR CONTRIBUTIONS

**Eugenia Volkova:** Conceptualization (equal); data curation (lead); formal analysis (lead); investigation (lead); methodology (lead); writing – original draft (lead); writing – review and editing (equal). **Linda Procell:** Data curation (supporting); formal analysis (supporting). **Lingyang Kong:** Data curation (supporting); formal analysis (supporting. **Lakshmi Santhanam:** Conceptualization (supporting); methodology (equal); supervision (supporting); writing – review and editing (equal). **Sharon Gerecht:** Conceptualization (equal); funding acquisition (lead); methodology (equal); resources (lead); supervision (lead); writing – original draft (equal); writing – review and editing (equal).

## CONFLICT OF INTEREST

The authors declare no competing interests.

### PEER REVIEW

The peer review history for this article is available at https://publons.com/publon/10.1002/btm2.10403.

## Supporting information


**Supplementary Figure 1** Combined middle age and early aging female Hif2a HT and Hif2a HO exhibit characteristics of pulmonary hypertension and erythrocytosis. (A) Red blood cell (B) Fulton index (C), normalized heart weight, (D) hematocrit, (E) hemoglobin, (F) white blood cell, (G) platelet counts, (H) immune cell concentrations including neutrophil (NE), lymphocyte (LY), monocyte (MO), eosinophil (EO), and basophil (BA). The data are presented as mean (*N* = 3–4). One‐way ANOVA was performed (Tukey's test for multiple comparisons). * indicates *p* < 0.05 and ** indicates *p* < 0.01 respectively.
**Supplementary Figure 2:** Combined middle age and early aging male Hif2a HT and Hif2a HO exhibit characteristics of pulmonary hypertension and erythrocytosis. (A) Red blood cell, (B) Fulton index, (C) normalized heart weight, (D) hematocrit, (E) hemoglobin, (F) white blood cell, (G) platelet counts, (H) immune cell concentrations including neutrophil (NE), lymphocyte (LY), monocyte (MO), eosinophil (EO), and basophil (BA). The data are presented as mean (*N* = 3–4). One‐way ANOVA was performed (Tukey's test for multiple comparisons). * indicates *p* < 0.05 and ** indicates *p* < 0.01 respectively.
**Supplementary Figure 3**: Sex differences in survival rates in Hif2a HT mice with aging. Kaplan–Meier survival analysis for WT and HT (A) female (*N* = 52) and (B) male (*N* = 49) mice.
**Supplementary Figure 4**: Female Hif2a HT mice exhibit compromised vascular mechanics as a consequence of aging‐dependent disease progression. (A) Tensile Curves of CTT of young adult (YA, 8–16 weeks) and middle age (M, 9–12 months) WT Female Thoracic Aorta Segments. (*N* = 6–11, *n* = 2–4). (B) Tensile Curves of CTT of young adult (YA, 8–16 weeks) and middle age (M, 9–12 months) Hif2a HT Female Thoracic Aorta Segments. (*N* = 5–10, *n* = 2–4). (C) Tensile Curves of CTT of young adult (YA, 8–16 weeks) and middle age (M, 9–12 months) WT Male Thoracic Aorta Segments. (*N* = 6–7, *n* = 2–4). (D) Tensile Curves of CTT of young adult (YA, 8–16 weeks) and middle age (M, 9–12 months) Hif2a HT Male Thoracic Aorta Segments. (*N* = 7, *n* = 2–4).
**Supplementary Figure 5**: Sample Collagen III+ Quantification. (A) Original 40x image of WT female thoracic aorta stained for Collagen III and (B) Corresponding thresholding for Collagen III+ regions.
**Supplementary Figure 6**: High‐magnification quantification of elastin layers does not reveal statistically significant differences in morphology in female mice. Quantification of (A) Number of elastin lamellae, (B) interlamellar distance, and (C) lamellar thickness (*N* = 3–7). A one‐way ANOVA (Tukey's Test for multiple comparisons) was performed. No significance found.

## Data Availability

The data that support the findings of this study are available from the corresponding author upon reasonable request.
