# Supplementary material for: Vascular stiffening in aging females with a hypertension‐induced HIF2A gain‐of‐function mutation
Source: Bioeng Transl Med. 2022 Oct 3;8(2):e10403. doi: 10.1002/btm2.10403 (PMC10013765; doi:10.1002/btm2.10403)
Supplement: Supplementary file 1 — Supplementary Figure 1 Combined middle age and early aging female Hif2a HT and Hif2a HO exhibit characteristics of pulmonary hypertension and erythrocytosis. (A) Red blood cell (B) Fulton index (C), normalized heart weight, (D) hematocrit, (E) hemoglobin, (F) white blood cell, (G) platelet counts, (H) immune cell concentrations including neutrophil (NE), lymphocyte (LY), monocyte (MO), eosinophil (EO), and basophil (BA). The data are presented as mean (N = 3–4). One‐way ANOVA was performed (Tukey's test for multiple comparisons). * indicates p < 0.05 and ** indicates p < 0.01 respectively. Supplementary Figure 2: Combined middle age and early aging male Hif2a HT and Hif2a HO exhibit characteristics of pulmonary hypertension and erythrocytosis. (A) Red blood cell, (B) Fulton index, (C) normalized heart weight, (D) hematocrit, (E) hemoglobin, (F) white blood cell, (G) platelet counts, (H) immune cell concentrations including neutrophil (NE), lymphocyte (LY), monocyte (MO), eosinophil (EO), and basophil (BA). The data are presented as mean (N = 3–4). One‐way ANOVA was performed (Tukey's test for multiple comparisons). * indicates p < 0.05 and ** indicates p < 0.01 respectively. Supplementary Figure 3: Sex differences in survival rates in Hif2a HT mice with aging. Kaplan–Meier survival analysis for WT and HT (A) female (N = 52) and (B) male (N = 49) mice. Supplementary Figure 4: Female Hif2a HT mice exhibit compromised vascular mechanics as a consequence of aging‐dependent disease progression. (A) Tensile Curves of CTT of young adult (YA, 8–16 weeks) and middle age (M, 9–12 months) WT Female Thoracic Aorta Segments. (N = 6–11, n = 2–4). (B) Tensile Curves of CTT of young adult (YA, 8–16 weeks) and middle age (M, 9–12 months) Hif2a HT Female Thoracic Aorta Segments. (N = 5–10, n = 2–4). (C) Tensile Curves of CTT of young adult (YA, 8–16 weeks) and middle age (M, 9–12 months) WT Male Thoracic Aorta Segments. (N = 6–7, n = 2–4). (D) Tensile Curves of CTT of young adult (YA [file BTM2-8-e10403-s001.docx]

**Vascular stiffening in aging females with a hypertension-induced HIF2A gain-of-function mutation**

Eugenia Volkova^1,2^, Linda Procell^1^, Lingyang Kong^1^, Lakshmi Santhanam^3,4^, Sharon Gerecht^1,2,4,5^

**Supplementary Figures:**


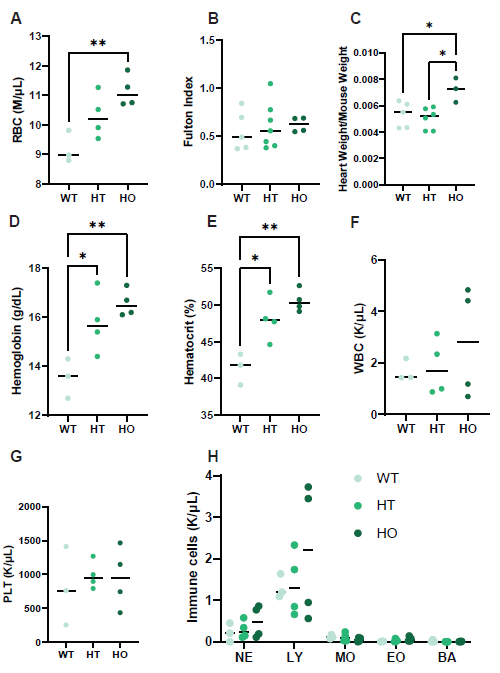


**Supplementary Figure 1: Combined middle age and early aging female Hif2a HT and Hif2a HO exhibit characteristics of pulmonary hypertension and erythrocytosis. (A)** Red Blood Cell **(B)** Fulton Index **(C)** Normalized Heart Weight **(D)** Hematocrit **(E)** Hemoglobin **(F)** White Blood Cell **(G)** Platelet counts **(H)** Immune cell concentrations including Neutrophil (NE), Lymphocyte (LY), Monocyte (MO), Eosinophil (EO, and Basophil (BA). The data are presented as means (N=3-4). One-way ANOVA was performed (Tukey’s test for multiple comparisons).

^*^ indicates p < 0.05 and ^**^ indicates p < 0.01 respectively.


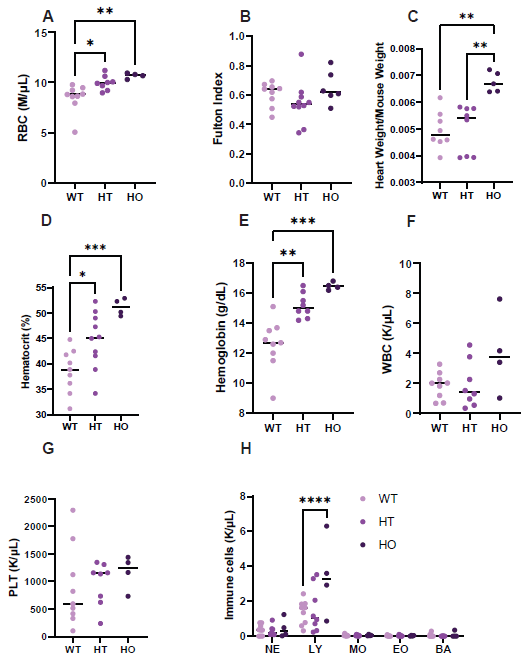


**Supplementary Figure 2: Combined middle age and early aging male Hif2a HT and Hif2a HO exhibit characteristics of pulmonary hypertension and erythrocytosis. (A)** Red Blood Cell **(B)** Fulton Index **(C)** Normalized Heart Weight **(D)** Hematocrit **(E)** Hemoglobin **(F)** White Blood Cell **(G)** Platelet counts **(H)** Immune Cell concentrations including Neutrophil (NE), Lymphocyte (LY), Monocyte (MO), Eosinophil (EO, and Basophil (BA). The data are presented as means (N=3-4). One-way ANOVA was performed (Tukey’s test for multiple comparisons).

^*^ indicates p < 0.05 and ^**^ indicates p < 0.01 respectively.


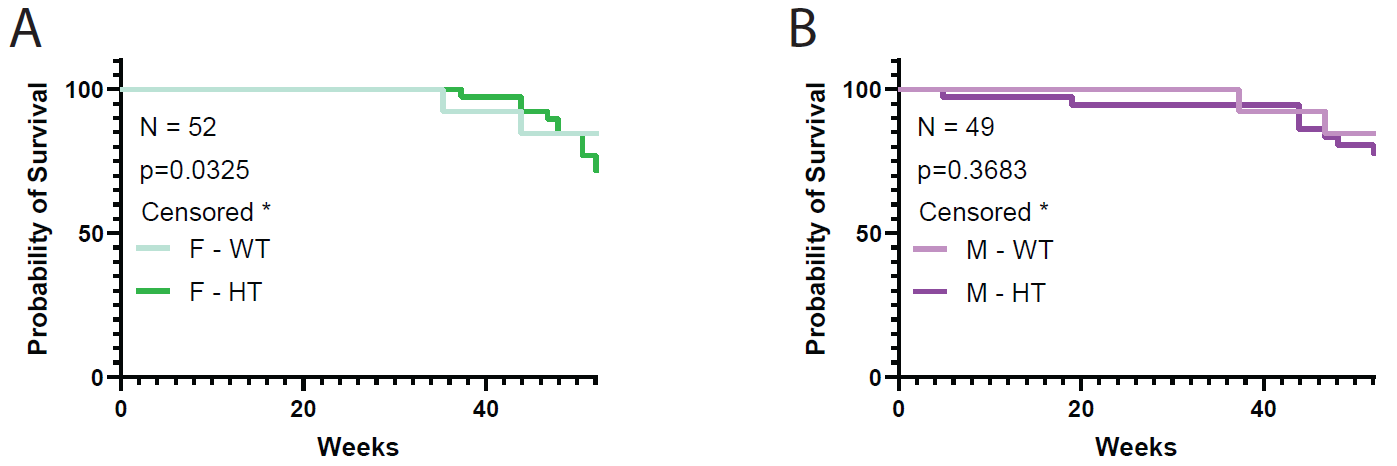


**Supplementary Figure 3: Sex differences in survival rates in Hif2a HT mice with aging.**

Kaplan-Meier survival analysis for WT and HT (A) female (N=52) and (B) male (N=49) mice.


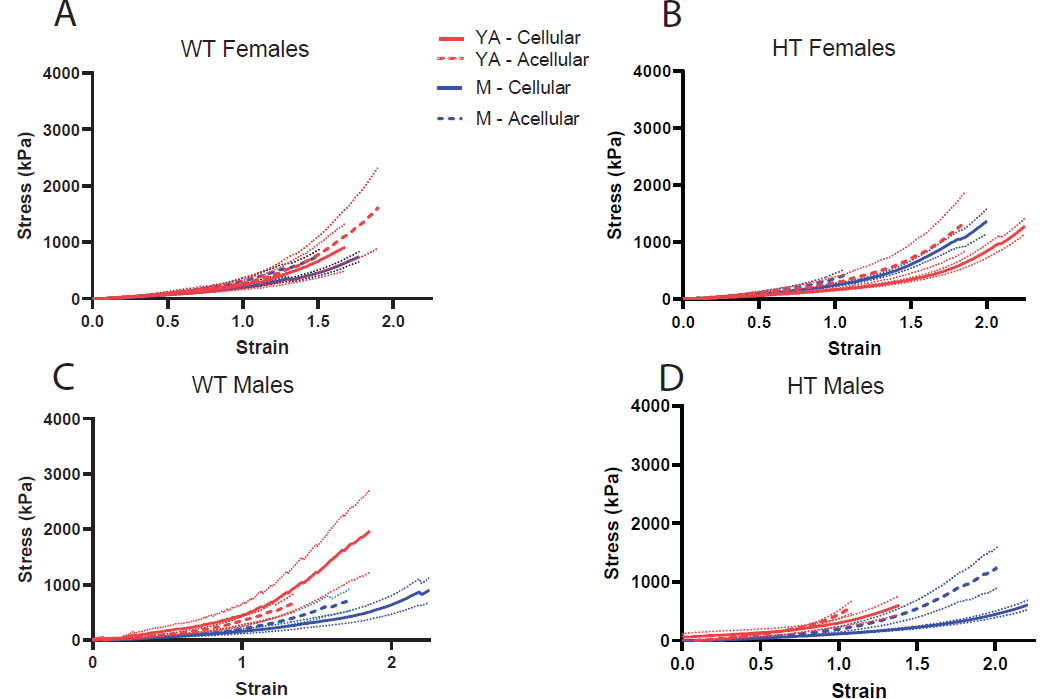


**Supplementary Figure 4: Female Hif2a HT mice exhibit compromised vascular mechanics as a consequence of aging-dependent disease progression. (A)** Tensile Curves of CTT of young adult (YA, 8-16 weeks) and middle age (M, 9-12 months) WT Female Thoracic Aorta Segments. (N = 6-11, n = 2-4). **(B)** Tensile Curves of CTT of young adult (YA, 8-16 weeks) and middle age (M, 9-12 months) Hif2a HT Female Thoracic Aorta Segments. (N = 5-10, n = 2-4). **(C)** Tensile Curves of CTT of young adult (YA, 8-16 weeks) and middle age (M, 9-12 months) WT Male Thoracic Aorta Segments. (N = 6-7, n = 2-4). **(D)** Tensile Curves of CTT of young adult (YA, 8-16 weeks) and middle age (M, 9-12 months) Hif2a HT Male Thoracic Aorta Segments. (N = 7, n = 2-4).


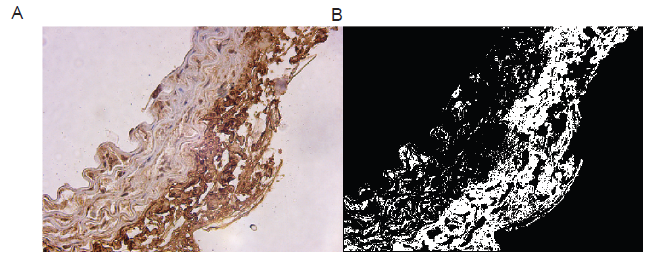


**Supplementary Figure 5: Sample Collagen III+ Quantification. (A)** Original 40x image of WT female thoracic aorta stained for Collagen III and **(B)** Corresponding thresholding for Collagen III+ regions.


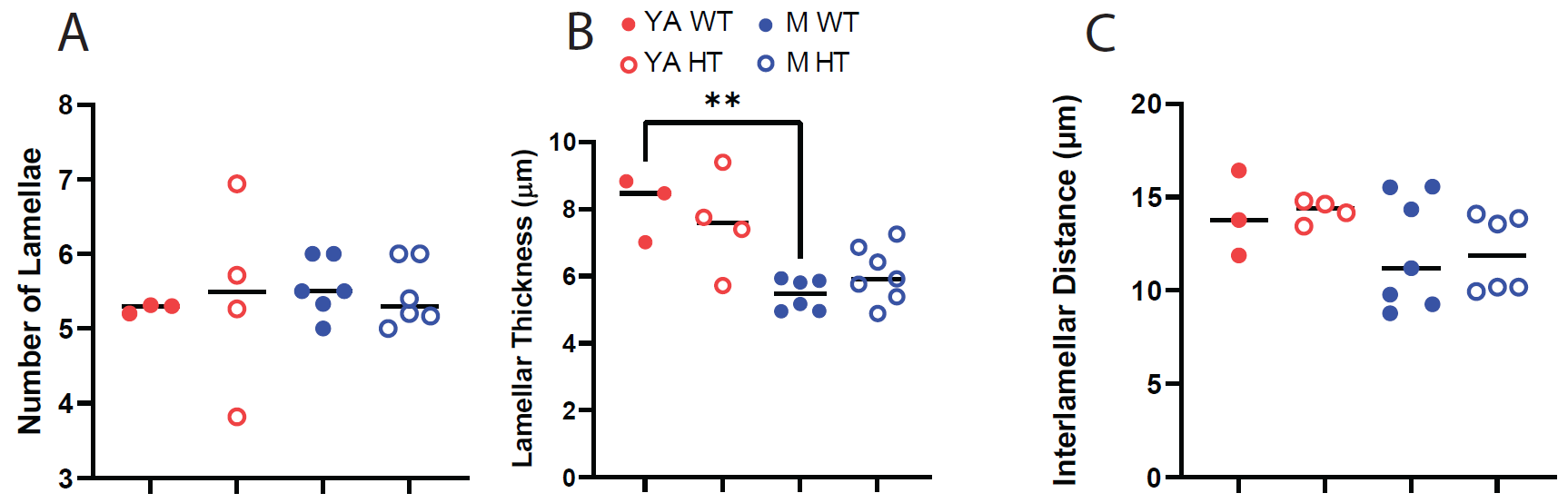


**Supplementary Figure 6: High-magnification quantification of elastin layers does not reveal statistically significant differences in morphology in female mice.** Quantification of **(A)** Number of elastin lamellae, **(B)** interlamellar distance, and **(C)** lamellar thickness (N=3-7). A one-way ANOVA (Tukey’s Test for multiple comparisons) was performed. ^**^ indicates p < 0.01
